# Synergy between tuberculin skin test and proliferative T cell responses to PPD or cell-membrane antigens of *Mycobacterium tuberculosis* for detection of latent TB infection in a high disease-burden setting

**DOI:** 10.1371/journal.pone.0204429

**Published:** 2018-09-24

**Authors:** Suvrat Arya, Shashi Kant Kumar, Alok Nath, Prerna Kapoor, Amita Aggarwal, Ramnath Misra, Sudhir Sinha

**Affiliations:** 1 Department of Clinical Immunology, Sanjay Gandhi Postgraduate Institute of Medical Sciences, Lucknow, India; 2 Department of Pulmonary Medicine, Sanjay Gandhi Postgraduate Institute of Medical Sciences, Lucknow, India; 3 DOT Centre, Sanjay Gandhi Postgraduate Institute of Medical Sciences, Lucknow, India; Indian Institute of Technology Delhi, INDIA

## Abstract

Tuberculin skin test (TST) is used most widely for the detection of latent tuberculosis infection (LTBI), even though evidences suggest that it could be underreporting the prevalence of LTBI particularly in high disease-burden settings. We have explored whether *in vivo* (TST) and *in vitro* (cell-proliferative) T cell responses to PPD can serve as complementary measures. In addition, we also probed whether *in vitro* T cell response to cell-membrane antigens (Mem) of *Mycobacterium tuberculosis* (MTB) can serve as a biomarker for LTBI. Study subjects comprised 43 healthcare workers (HCWs), and 9 smear-positive TB patients served as ‘disease control’. To measure proliferative T cell responses, 0.1 ml blood (diluted 1:10) was incubated (5 days) with test or control antigen. Cells were stained with fluorescent antibodies to T cell (CD3+/CD4+/CD8+) surface markers and, after fixation and permeabilization, to nuclear proliferation marker Ki67. Data was acquired on a flow cytometer. HCWs who had an intimate exposure to MTB showed significantly higher TST positivity (85%) than the rest (43%), notwithstanding their BCG vaccination status. The proliferative responses of CD4+ and CD8+ subsets of T cells were comparable. Sixty seven and 100% TST-negative HCWs, respectively, were positive for proliferative T cell response to PPD and MTBMem. Cumulative positivity (TST or *in vitro*) was 86% with PPD and 100% with MTBMem indicating complementarity of the two responses. As standalone *in vitro* assay, MTBMem provided a significantly higher positivity (95%) than PPD (67%). T cell responses of TB patients were ‘generally’ depressed, having implications for the development of immunological assays for ‘progressive’ LTBI. Altogether, these results demonstrate that *in vivo* and *in vitro* T cell responses to PPD are complementary and *in vitro* response to MTBMem can be developed as a highly sensitive biomarker for LTBI.

## Introduction

A vast majority of persons living in high tuberculosis (TB) burden countries harbor latent TB infection (LTBI), defined as ‘a state of persistent immune response to *Mycobacterium tuberculosis* (MTB) without clinically manifested disease’ [1]. Approximately 10% individuals with LTBI may develop active TB over a span of 2–5 years [2]. In 2016, 6.3 million new TB patients were reported worldwide against the estimated 10.4 million, implying that nearly 4 million cases were ‘missing’ from records [2]. India alone accounts for a quarter of the missing cases who could be acting as ‘hidden’ foci of infection. Detection of MTB infection, preferably at a ‘preclinical’ stage, is considered essential to the success of ‘End TB’ strategy [2].

In absence of a ‘gold standard’, the most widely used test for detection of LTBI is ‘Tuberculin Skin Test (TST)’. A major criticism of TST is that the test results are confounded by prior vaccination with BCG or exposure to the ‘non-tuberculous’ mycobacteria (NTM). The available data, however, point to the contrary. In a meta-analysis of 24 studies involving 240,203 subjects, TST positivity was seen in <1% of subjects who were BCG vaccinated during infancy and tested 10 years later [3]. In the same meta-analysis, NTM also was not found to be a significant confounder of TST. In 18 studies involving 1,169,105 subjects, false-positive TST due to NTM ranged from 0.1 to 2.3%. Therefore, in countries such as India which follow the WHO policy of BCG vaccination during infancy [2] a TST response of ≥10 mm in adolescents and adults can be considered as a reliable indicator of MTB infection [4, 5]. Head-to-head comparisons of TST with ‘Interferon Gamma Release Assays (IGRAs)’ have shown no evidence that one test is better than the other [1]. In fact, contrary to the expectation, some studies have found TST to have an edge over IGRA [6, 7].

Though TST and IGRA can detect MTB infection with reasonable specificity, both apparently fall short of a desirable sensitivity as they often fail to detect even bacteriologically proven cases of TB [8]. This raises the concern as to whether these assays are sensitive enough to detect LTBI. In a recent study [9], performances of TST and IGRA were evaluated in over 1500 contacts of TB from Delhi. 76 contacts developed active TB during the follow-up. Strikingly, incidence of cases from TST or IGRA positive and negative contacts was comparable, suggesting that both tests could be missing a large proportion of MTB-infected subjects living in high disease-burden areas.

Considering that circulating T cells may display a broader phenotypic diversity than those in the skin, studies were undertaken to explore whether blood T cells of TST-negative individuals will respond to PPD. Indeed, PPD could induce T cell proliferation in TB patients who were TST non-responders [10]. PPD (and other MTB antigens) also induced IFN-γ synthesis in T cells of TST-negative healthy individuals from TB-endemic areas [11, 12]. These reports suggest that *in vivo* (TST) and *in vitro* T cell responses to PPD can complement each other. Further, a genome-wide search has identified 82 MTB proteins containing immunodominant epitopes for T cells of LTBI-positive subjects [13]. Interestingly, ESAT-6 and CFP-10 (key components of IGRA) did not figure among those antigens which comprised mostly the membrane-associated proteins of MTB. Immunodominance of mycobacterial membrane proteins for human T and B cells has also been reported by us [14, 15] as well as others [16, 17].

The ongoing TB epidemic is driven by a massive increase in case notifications from India [2]. Also, a recent report of IUATLD reveals that India has the highest incidence of child TB [18]. These data imply that the true expanse of LTBI or ‘annual risk of TB infection (ARTI)’ in India could be much higher than the estimates which are based almost entirely on TST surveys [4, 19]. Remarkably, most surveys have reported similar rates of TST positivity (~50%) in general population and close contacts of TB [20, 21], even though individuals in the latter category stand a higher risk of acquiring the infection [9, 22]. Our aim in this study was to explore whether (a) *in vitro* and *in vivo* T cell responses to PPD can serve as complementary measures of LTBI and (b) *in vitro* T cell response to cell-membrane antigens of MTB can be harnessed as a sensitive biomarker for LTBI. Study subjects were healthcare workers (HCWs) of a large tertiary-care hospital in north India, and the ‘disease control’ comprised sputum-smear positive TB patients. Several studies [2], including from India [23], have reported that HCWs stand a greater risk of acquiring MTB infection than the population-at-large.

## Materials and methods

### Study setting

The study was conducted at Sanjay Gandhi Postgraduate Institute of Medical Sciences (SGPGIMS), which is a large (>1000 beds) tertiary referral medical school in the city of Lucknow, located in northern India. The DOT clinic of SGPGIMS treats approximately 300 TB patients annually.

### Study subjects

The study group comprised 43 HCWs (faculty, students and staff) of SGPGIMS (Table 1 under Results). In addition, 9 pulmonary TB patients (3 female, 6 male, 23–55 Y) were included as ‘disease control’. All patients had active disease with sputum-smears positive (2+ to 3+) for acid-fast bacilli (AFB). Six of them were drug naïve and remaining 3 had received <3 weeks of multi-drug therapy [2]. None of the study subjects was suspected of being infected with, or had tested positive for, HIV. Study protocol was approved by the Institutional Ethics Committee of Sanjay Gandhi Postgraduate Institute of Medical Sciences and all participants provided written informed consent. All clinical investigations were conducted as per guidelines of the ‘Helsinki Declaration’ [24].

**Table 1:** Demographic data of study subjects.

Category	Number (Female)	Age, Years (Median)	BCG Scar+ (%)	TST Positive (%)
OC*	30 (5)	19–56 (33)	25 (83)	13 (43)
HC**	8 (1)	20–61 (34)	6 (75)	7 (88)
Cured TB	5 (2)	28–61 (40)	4 (80)	4 (80)
TOTAL	43 (8)	19–61 (33)	35 (81)	24 (56)

* Occupational contact.

** Household contact.

## Materials

Details of purchased materials are given under supporting information (S1 Text).

### Preparation of bacterial cell membrane antigens

Membranes of MTB (MTBMem) and *E*. *coli* were isolated by using a previously described protocol [14], with some modifications. In brief, 3 week old cultures of MTB (on Lowenstein-Jensen medium) or 24 h old cultures of *E*. *coli* (in Luria-Bertani broth) were harvested and bacterial sediments washed and suspended in PBS (0.2 g wet wt/ ml). The cell lysates, obtained by sonication (20 min), were centrifuged (23,000g x 20 min) to settle unbroken cells and cell-wall debris. The supernatants were re-centrifuged (150,000g x 90 min) to obtain membrane (sediment) and cytosol (supernatant). Membranes were washed and reconstituted with PBS. Protein estimations were done by modified Lowry method [25] and protein profiles were determined by SDS-PAGE (S1 Fig). Suitable aliquots were stored at -80°C.

### TST

Five tuberculin units (TU) of PPD (0.1 ml) were injected intradermally on the volar aspect of left forearm. After 48–72 hours, maximum diameter of palpable induration was measured using a calliper [26]. TST was considered as positive if induration was 10 mm or more.

### Blood samples

Blood was collected in sodium heparin tubes by standard venipuncture. Samples were kept at room temperature (RT) until used for setting up the assays (within 4 h of collection).

### T cell proliferation assay

Blood was diluted (1:10) in RPMI medium and dispensed in 24-well culture plates (1 ml/ well). To individual wells, medium (negative control), PHA (5 μg, positive control) or test antigens- PPD (5 TU or 0.1 μg) and MTBMem (5 μg) were added and incubated for 5 days in a CO_2_ incubator. For some assays, *E*. *coli* membrane (5 μg) was also used as ‘negative’ control. On day-5, the cells were treated with EDTA (2 mM, 15 min), collected in FACS tubes and centrifuged (400g x 5 min). To the vortexed cell sediments, predetermined quantities of fluorescent-tagged antibodies to T cell surface markers (CD3/CD4/CD8) were added and incubated (30 min, RT, in dark). RBCs were lysed by BD FACS lysing solution (2 ml/tube, 15 min, RT, in dark) followed by centrifugation. WBCs (sediment), washed with PBS, were fixed (15 min, RT, in dark) with 2% para-formaldehyde. Later, cells were washed with PBS containing 0.05% BSA (PBS-BSA) and permeabilized (15 min, RT, in dark) with 0.2% Triton X100. Cells were again washed with PBS-BSA and incubated (45 min, RT, in dark) with fluorescent-tagged antibody to Ki67 (a nuclear protein, overexpressed during DNA synthesis) [27]. Cells were finally washed with PBS-BSA and resuspended in PBS. Data on 10^5^ cells in ‘lymphocyte gate’ was acquired on a FACS Canto-II flow cytometer (BD) and analysed using Flowjo software v10.0.7 (Tree Star, USA). Cut-off for a positive cell-proliferative response was determined as 0.75% (= mean + 3 x SD of all responses to RPMI medium).

Gating strategy is depicted in S2 Fig. Additional data on method optimization is given in S1 File.

### Statistical analysis

Student’s t test (paired/unpaired) was used to compute the differences between two populations and Fisher’s exact test (using 2x2 contingency tables) was used to compute differences between two proportions. P values <0.05 were considered as significant. Statistical analyses were performed using GraphPad Prism software.

## Results

### Study subjects

Demography of study subjects is summarised in Table 1. Of the 43 HCWs, 30 had not lived with a TB patient in their households and were classified as ‘occupational contacts (OC)’. Eight HCWs, who had lived with a bacteriologically positive TB patient for at least 3 months, were classified as ‘household contacts (HC)’. Five HCWs had a history of cured TB (2 pulmonary, 2 lymphadenitis and 1 cutaneous). BCG scar was present in 81% subjects and 56% tested positive for TST.

### TST responses reflected exposure to MTB independently of BCG vaccination

**Fig 1:**
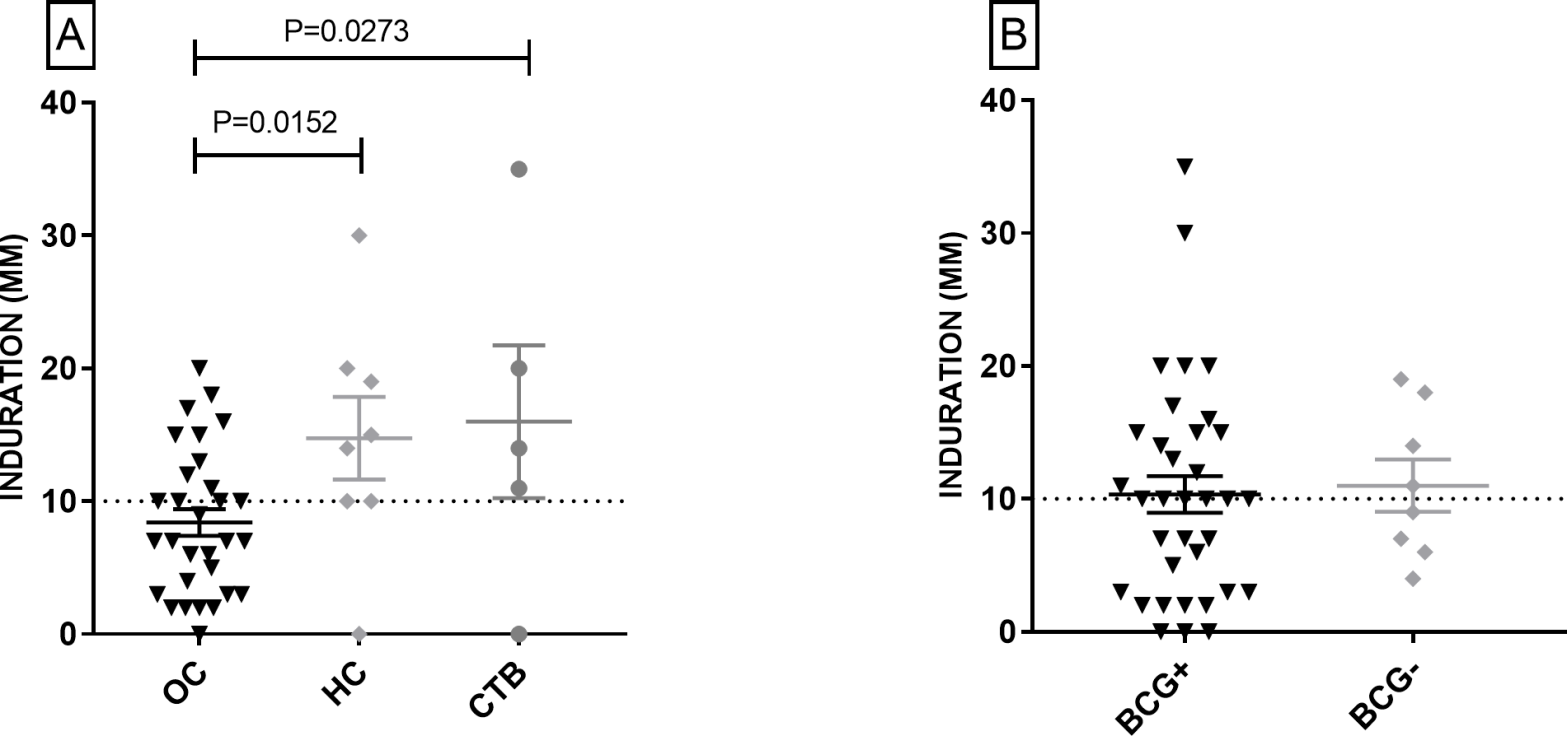
Response of HCWs to TST. [A] Responses (skin induration) of household contacts (HC) and cured TB patients (CTB) were significantly stronger than the occupational contacts (OC), and [B] responses of subjects with (+) or without (-) BCG scar were comparable. Mean +/- SEM of values in each column are depicted and *P* values are shown on top of corresponding columns. Dashed horizontal lines denote cut-off for a positive response (= 10 mm induration).

As shown in Table 1 (and S1 Table), 43% subjects in the OC category (n = 30) were positive for TST. However, rate of positivity (85%) was significantly higher (P <0.05) in HCWs who had been exposed to or infected with MTB (HC and cured TB, n = 13). Skin indurations in these subjects were also significantly larger than OC (Fig 1A, S1 Table). On the other hand, rates of TST positivity in HCWs who had (57%, 20/35) or had not (50%, 4/8) received the BCG vaccine (determined by the presence of BCG scar) did not differ significantly. Skin indurations in both groups were also comparable (Fig 1B, S2 Table). These results suggested that individual TST responses could reflect the exposure to MTB, independently of the BCG vaccine taken during infancy.

### MTB antigens induced proliferative responses in CD4+ as well as CD8+ subsets of T cells

**Fig 2:**
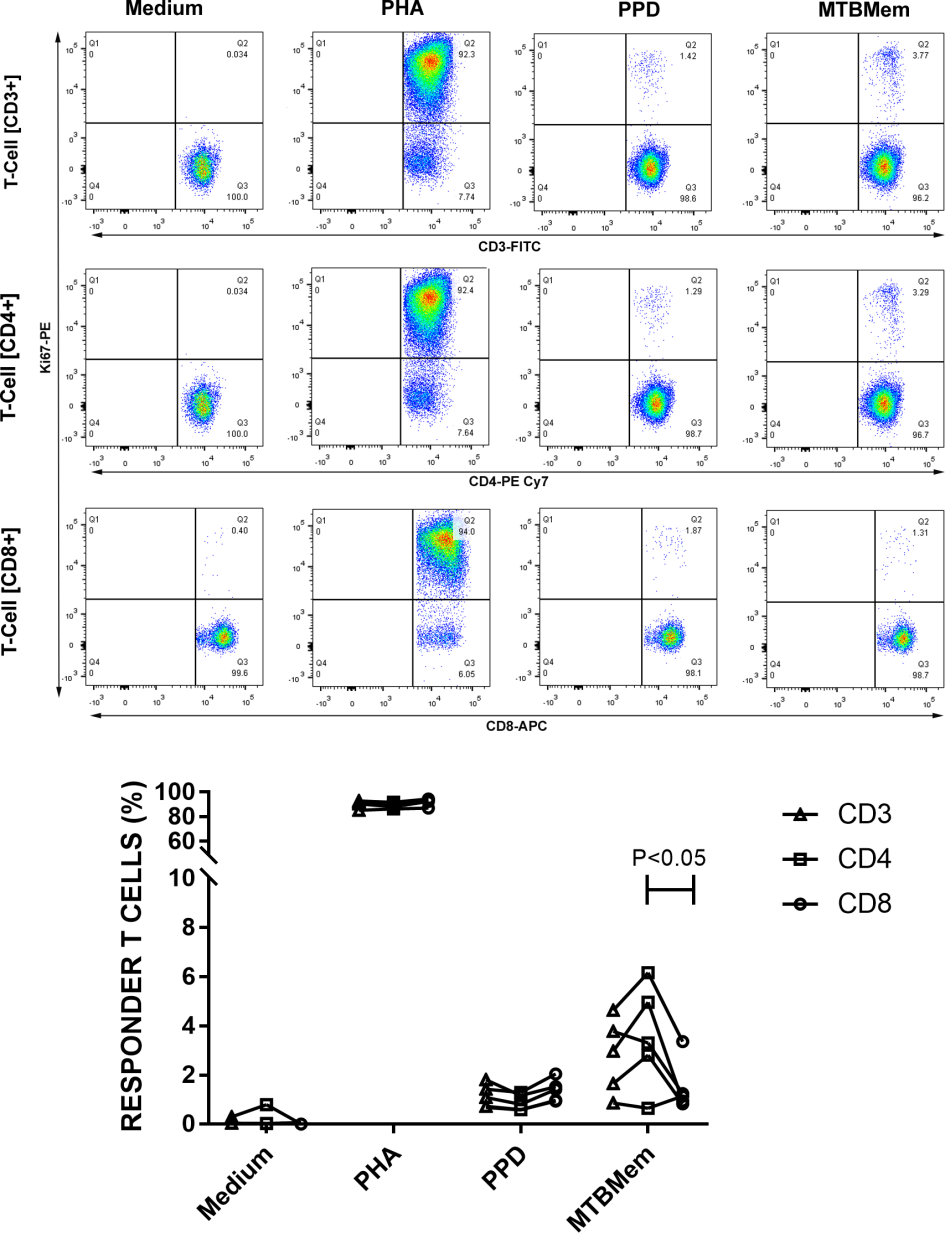
Proliferative responses of T cells (CD3+) and T cell subsets (CD4+ and CD8+) to MTB antigens (PPD and MTBMem) or controls (medium and PHA). Top Panel: Representative flow plots for one of the HCWs. In each plot, upper right quadrant houses the responder (Ki67+) cells. Bottom Panel: Individual T cell responses (key) of 5 HCWs. *P* value for the lone difference is shown.

We initially compared the proliferative responses of CD4+ and CD8+ cells to PPD and MTBMem antigens since both subsets of T (CD3+) cells contribute to overall T cell-mediated immunity against MTB [28]. Both showed comparable responses to PPD and PHA (a T cell mitogen), though the response to MTBMem differed significantly (P <0.05, Fig 2 and S3 Table). These results and corroborative evidences [29] prompted us to adopt T cell proliferation as an indicator of the T cell response. These results also pointed to a higher T cell stimulatory potency of MTBMem than PPD, which was a consistent feature of the study.

### *In vivo* and *in vitro* T cell responses to PPD were complementary

**Fig 3:**
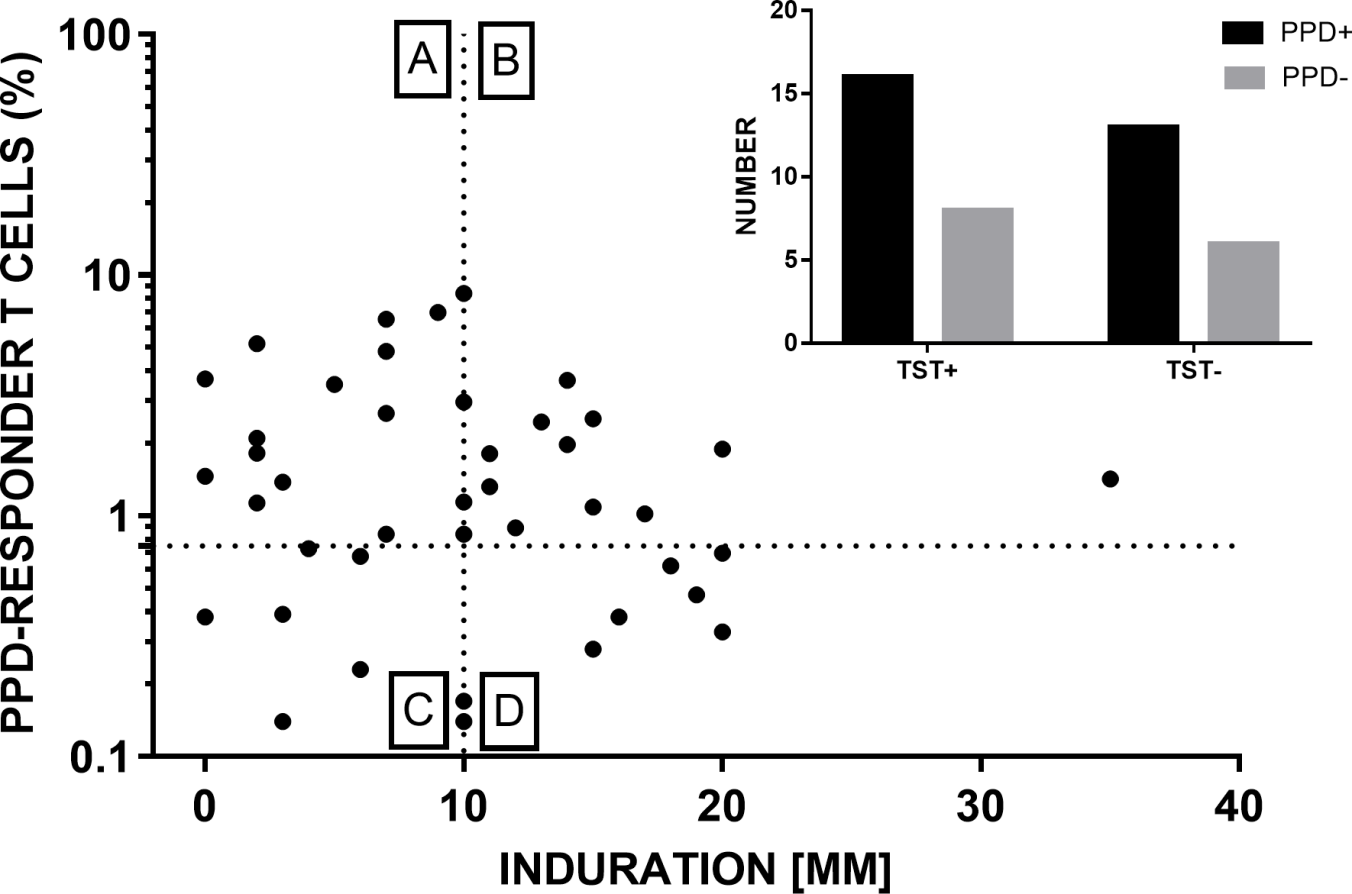
Complementarity of *in vivo* and *in vitro* T cell responses to PPD. Cutoffs for positive skin reaction (10 mm induration) and cell proliferation (0.75% responder cells) are shown by dashed lines. Quadrants A-D contain subjects who were [A] negative for *in vivo* but positive for *in vitro* response, [B] positive for both responses, [C] negative for both responses, and [D] positive for *in vivo* but negative for *in vitro* response. Inset figure (S5 Table) shows absolute numbers in each category.

Proliferative T cell responses of HCWs to PPD, along with corresponding TST indurations are depicted in Fig 3 (and S4 Table). Positivity for the *in vivo* (TST) response was 56% (Table 1) and that for *in vitro* response was 67% (29/43). Cumulative positivity (for either assay) was 86% (37/43), which was significantly higher (P <0.01) than positivity for TST alone. Subjects who were non-responders to PPD (quadrant C, Fig 3) belonged mostly to the OC category. These results indicated that *in vivo* and *in vitro* T cell responses to PPD could serve as complementary measures.

### MTB membrane was a potent activator of mycobacterium-specific T cells

**Fig 4:**
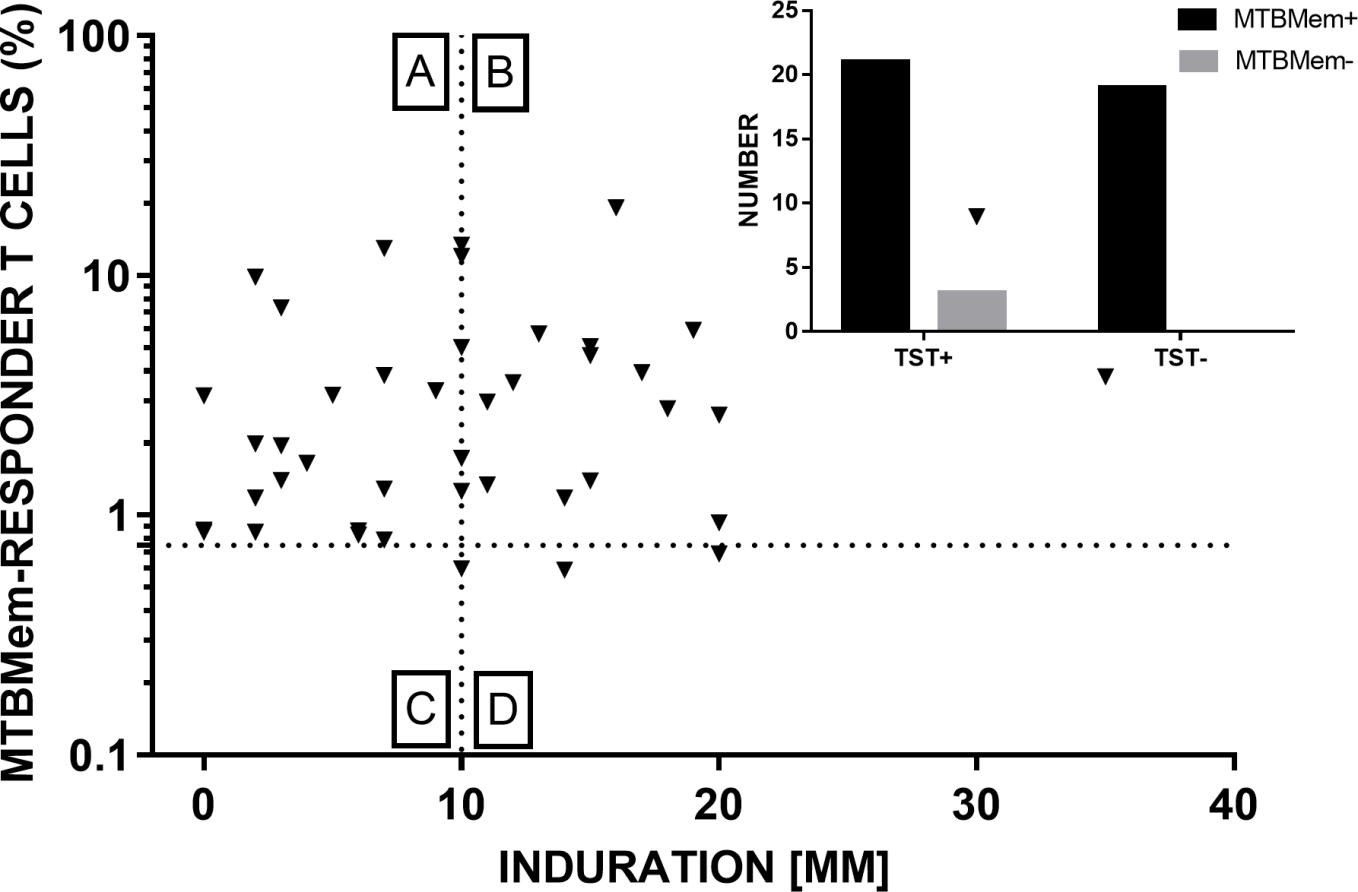
Complementarity of TST and *in vitro* T cell responses to MTBMem. Cutoffs for positive skin reaction (10 mm induration) and cell proliferation (0.75% responder cells) are shown by dashed lines. Quadrants A-D contain subjects who were [A] negative for *in vivo* but positive for *in vitro* response, [B] positive for both responses, [C] negative for both responses, and [D] positive for *in vivo* but negative for *in vitro* response. Inset figure (S6 Table) shows absolute numbers in each category.

**Fig 5:**
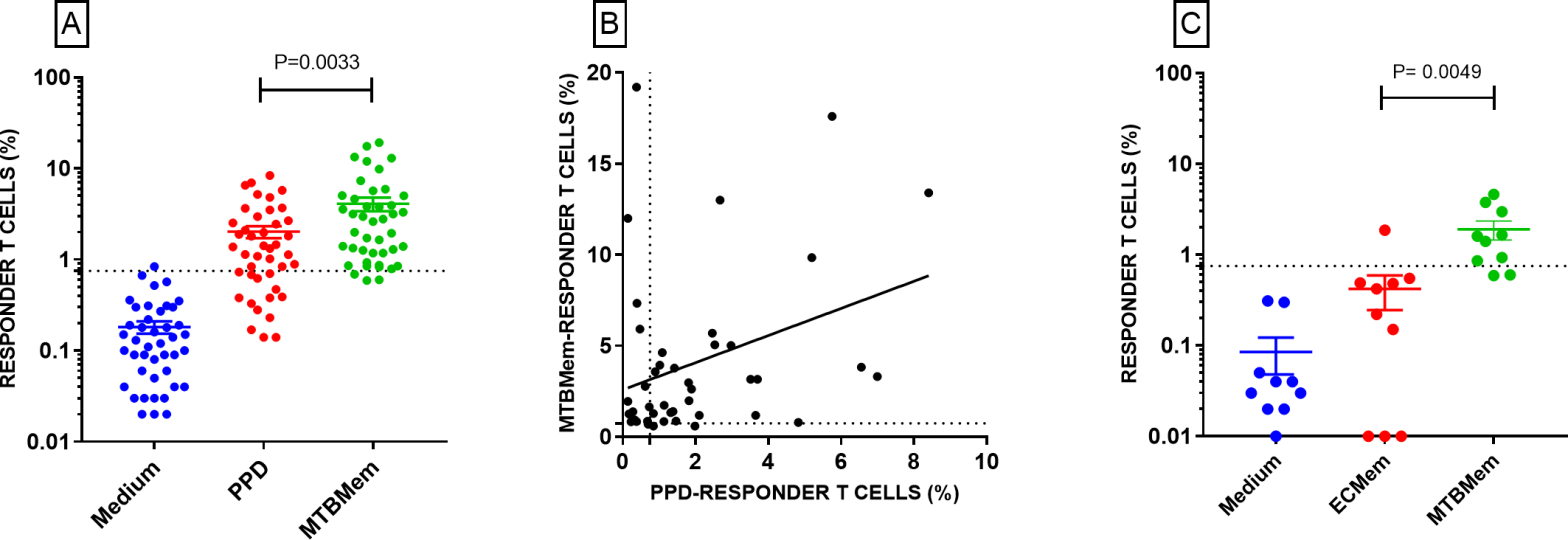
Relative potency and specificity of the T cell response induced by MTBMem. Dashed lines denote cutoff for a positive cell-proliferative response (= 0.75% responder cells). [A] MTBMem produced significantly higher (P <0.01) response than PPD. [B] A weak (r = 0.33) though significant (P <0.05) correlation was seen between responses to MTBMem and PPD. [C] MTBMem also produced a significantly higher (P <0.01) response than *E*. *coli* membrane (ECMem).

A positive T cell proliferative response to MTBMem was seen in 88% (21/24) of TST positive and 100% (19/19) of TST negative HCWs (Fig 4 and S4 Table). Positivity for either assay (TST/*in vitro*) was 100%. As an antigen for ‘standalone’ *in vitro* assay, MTBMem produced a significantly higher (P <0.01) positivity (95%, Fig 4) than PPD (67%, Fig 3). Abundance of MTBMem-reactive T cells in HCWs was also significantly higher than PPD-reactive ones (Fig 5A). A weak, though significant, correlation (r = 0.33, P <0.05) between responses to both the antigens (Fig 5B) suggested that their unique proteins [14, 30] could have triggered T cells of diverse antigenic specificities. Altogether, these results indicated a stronger T cell inducing potency of MTBMem than PPD.

To assess the specificity of T cell response induced by MTBMem, we compared it with the response to *E*. *coli* membrane (Fig 5C, S7 Table). All but one proliferative response to *E*. *coli* antigen was negative and the difference between responses to the two antigens was significant (P <0.01). These results suggested that the T cell response induced by MTBMem was at least ‘mycobacterium-specific’, if not MTB-specific.

### T cell responses declined with increasing exposure to MTB

**Fig 6:**
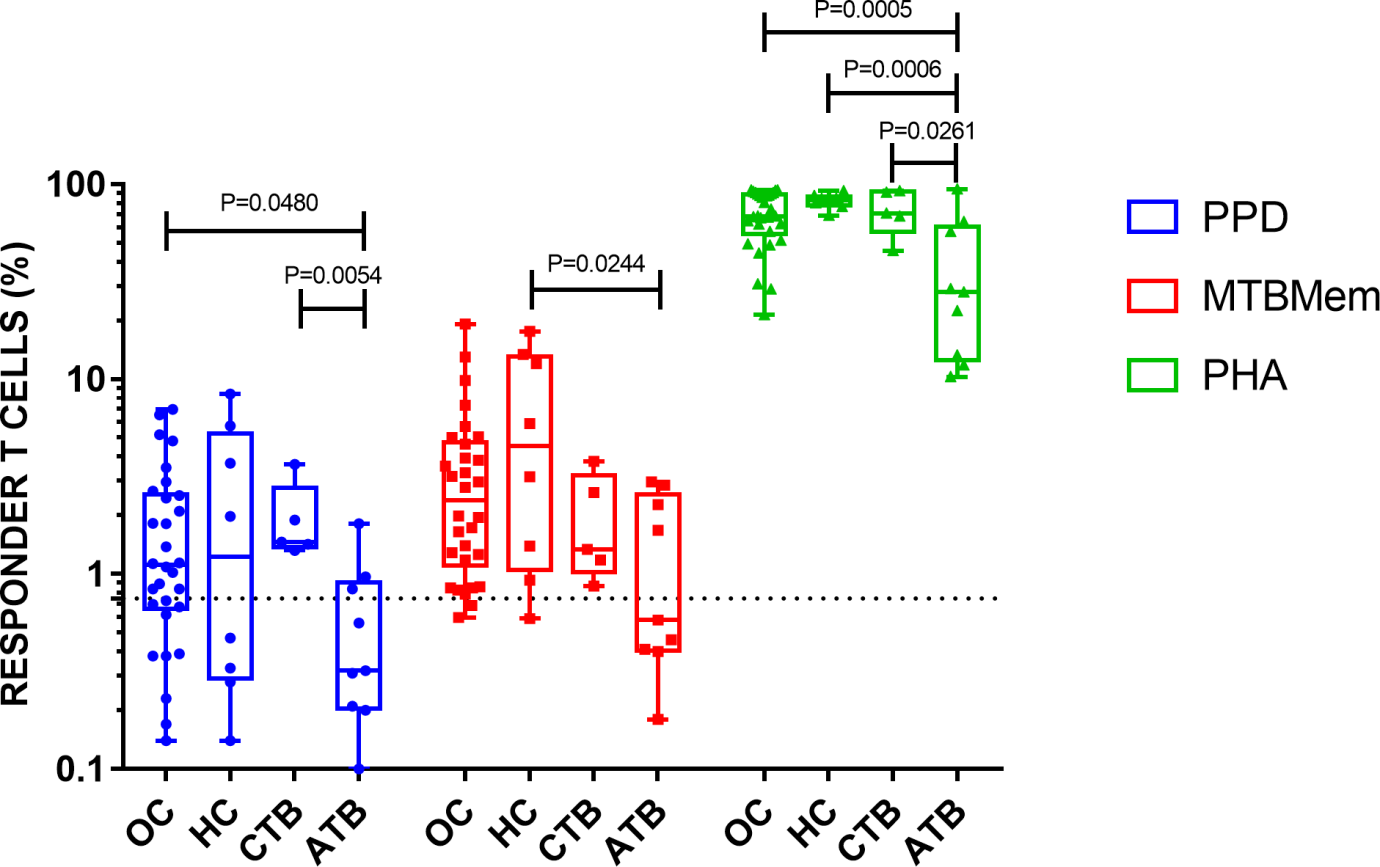
Proliferative T cell responses to MTB antigens and mitogen (key) declined with escalating exposure to the infection. Box and whisker plots show individual responses of occupational contacts (OC), household contacts (HC), cured TB (CTB) and active TB (ATB) patients. Cutoff for a positive response (0.75% responder cells) is depicted by dashed line. *P* values for significant differences are shown on the top of corresponding columns.

An interesting picture emerged when T cell responses of HCWs were compared with those of TB patients who served as ‘disease control’ for this study. Though the responses to MTB antigens were marginally higher in HC (than OC), they were lower in subjects with cured TB and, more so, in patients with active TB (Fig 6, S8 Table). In some instances, the decline in response attained statistical significance. Remarkably, the response of TB patients to the T cell mitogen (PHA) was also significantly lower than corresponding response of HCWs (in all 3 categories). These results suggested that an increasing burden of MTB antigen or infection in an individual could produce MTB-specific and, eventually, a ‘generalized’ depression of T cell responses.

## Discussion

TST is being used for the detection of LTBI for nearly a century and WHO also recommends that it need not be replaced by IGRA [1, 2]. Nonetheless, availability and quality of PPD for TST surveys remain as areas of concern [31]. Another area of concern is wide variation in the used ‘strength’ (TU) of PPD [4, 19–22]. These variables may have led to major discrepancies in the reported prevalence of LTBI [32]. In deciding 5 TU PPD as the test dose, we were guided by the recommendations of IUATLD and WHO [26] as well as some studies from the Indian subcontinent [22, 33]. Rate of TST positivity in our HCWs was comparable with that from other locations in India [20, 23]. Stratification of HCWs revealed that enhanced exposures to MTB could generate higher rates of TST positivity, which was consistent with reports from Delhi [22] and Karachi [33]. The positivity of HCWs for BCG scar was also comparable with other reports from India which, like us, did not observe any influence of BCG vaccine on TST results [9, 21].

Even though responsiveness to TST may increase with rising exposure to the infection, incident TB cases can emerge from TST-negative subpopulations [4, 9]. To determine if *in vitro* T-cell response to PPD could complement the *in vivo* (TST) response, we used the same antigen preparation for both the assays. Indeed, both assays appeared complementary as two-thirds of the TST-negative HCWs showed positivity for T cell proliferation. Conversely, one third of TST-positive subjects did not show the *in vitro* response. Similar discordant *in vivo* and *in vitro* responses have been reported earlier also [11, 12]. Due to various reasons, people may show persistent unresponsiveness to TST despite living in TB hyperendemic areas. For instance, lack of expression of ‘cutaneous lymphocyte antigen’ on skin-resident T cells has been held responsible for a lack of TST response [34]. Deficiency in production of macrophage migration inhibitory factor (MIF) by skin macrophages may also contribute to poor TST responses [35]. The most likely reason behind absence of proliferative response in a subset of TST-positive HCWs could be overproduction of TGF-β in cell cultures, induced by PPD [36]. As a ‘master regulator’ of immune responses, TGF-β can suppress proliferative T cell response on one hand and, on the other, it may facilitate TST response by promoting maturation of the skin-resident memory T cells and antigen-presenting dendritic cells [37].

T cells of almost all HCWs, including TST-negative ones, responded to MTBMem. Even though constituents of mycobacterial cell-membrane are potent inducers of human T and B cells [13–17], such high positivity rates raise concern about specificity of the response. We therefore compared it with response to the membrane antigens of *E*. *coli*- a Gram negative bacterium that colonizes gastrointestinal and genitourinary tracts in humans. An insignificant T cell response to *E coli* membrane suggested that the response to MTBMem could be considered as ‘mycobacterium-specific’, while a deeper investigation (using antigens from other microbes and mycobacteria to which Indians are ‘environmentally’ exposed) is needed to prove that it is also MTB-specific. Comparable T cell responses of healthy Indians to MTB antigens have been reported earlier also. In a study from Mumbai [38], T cells of 80% volunteers responded to ESAT-6 and CFP-10 and the positivity level rose to 98% when PPD was used as antigen. Since a subset of subjects from Oxford also responded to PPD, the authors speculated that the latter response could be due to BCG. Nonetheless, given that influence of BCG wanes in later years of life [3], it is possible that the PPD-positive subjects were indeed harboring LTBI [5]. In another related study [39], 96% TB contacts were considered LTBI-positive as they harbored antibodies to ESAT-6 and CFP-10.

Our results with TB patients are consistent with reports showing depressed MTB-specific as well as general T-cell responses in heavily infected individuals. Though we did not perform TST in our patients (as their diagnosis could be established by smear microscopy), most TB patients with advanced disease are unresponsive to TST [33, 38, 40]. T-cell responses to IGRA antigens also decline with rising bacterial loads [29]. In the same study [29], impaired T-cell proliferation correlated positively with the frequency of polyfunctional (IFN-γ+IL-2+TNF-α+) and negatively with monofunctional (TNF-α+) T cells. Further, the authors observed a recovery in MTB-specific responses following chemotherapy. Cured TB cases in our study also showed higher T cell responses than active cases. A possible reason for depressed T-cell responses in active patients could be ‘compartmentalization’ of the T cells. Schwander, et al [41] have reported significantly higher PPD-induced synthesis of DNA and IFN-γ in bronchoalveolar cells than blood cells of TB patients. Another possible reason could be MTB-induced overproduction of the T-cell regulatory cytokine TGF-β in patients [36, 37]. These and our results have important implications for the development of T cell-based diagnostics against TB. While frequency of MTB-reactive T cells may be high in LTBI, it could decline as the infection progresses. This may make the distinction between ‘subclinical’ and ‘preclinical’ stages of infection a challenging proposition.

We strived to use methods and protocols which could be adopted for large-scale surveys, if required. T-cell proliferation is as good a correlate of immunity as the levels of multiple ‘type-1’ cytokines [29, 42]. The choice for whole blood over mononuclear cells jettisoned the need to draw larger volumes of blood and its ‘pre-processing’. Though we used 0.1 ml venous blood for each assay point it should be possible, with the advent of more sensitive and simpler versions of flow cytometers, to work with smaller volumes of capillary (finger prick) blood. The required laboratory setup is within the acceptable limits for a new TB diagnostic assay [43].

By definition, a test for LTBI provides evidence for memory immune response against MTB rather than confirming the presence of viable or active infection. With this perspective, our (and some earlier) results suggest that almost all residents of a TB hyper-endemic region could be considered as LTBI-positive. Then how to select those subjects who may progress towards active TB? The changing paradigm of LTBI as a ‘spectrum’ necessitates that any diagnostic test be performed at multiple time points [43]. For inclusion in such longitudinal surveys, it could be more useful to enrol those individuals who are not only test-positive at the baseline but also harbour a high frequency of MTB-reactive T cells. For example, against the positivity cut-off of 0.75%, a good number of our HCWs displayed 4 times higher (>3%) responder cells. In this context, large TST reactions have already been reported to be better predictors of incident TB disease [4].

A relatively small sample size could be considered as a limitation of the study. The applied criteria restricted us from recruiting larger number of subjects in the HC and cured TB categories. Similarly, the prevailing vaccination policies made it difficult to recruit more subjects in the BCG-negative category. In choosing TB patients also, our focus was on sputum-smear positive and treatment-naïve donors. Nonetheless, our results are well-supported by some prior studies [9, 11, 12, 20–23, 29, 33, 38, 40]. Other requirements, which may be met through future studies, include (a) data on performance of *in vitro* assays, especially using MTBMem, in regions with low TB endemicity and (b) molecular characterization of the antigens in MTBMem [13, 14]. To conclude, this study, conducted in the backdrop of an unabated TB epidemic, calls for a critical appraisal of methods and protocols currently in use for detection of LTBI.

## Supporting information

S1 Text Purchased materials. (DOCX)

S1 Fig Protein profiles of *M*. *tuberculosis* and *E*. *coli* cell membranes determined by SDS-PAGE. (DOCX)

S2 Fig Gating strategy for flow cytometry. (DOCX)

S1 File Optimization of T cell proliferation assay. (DOCX)

S1 Table Dataset for Fig 1A. (DOCX)

S2 Table Dataset for Fig 1B. (DOCX)

S3 Table Dataset for Fig 2B. (DOCX)

S4 Table Dataset for Figs 3, 4, 5A and 5B. (DOCX)

S5 Table Dataset for Fig 3 (inset). (DOCX)

S6 Table Dataset for Fig 4 (inset). (DOCX)

S7 Table Dataset for Fig 5C. (DOCX)

S8 Table Dataset for Fig 6. (DOCX)
