# Supplementary material for: Synergy between tuberculin skin test and proliferative T cell responses to PPD or cell-membrane antigens of Mycobacterium tuberculosis for detection of latent TB infection in a high disease-burden setting
Source: PLoS One. 2018 Sep 24;13(9):e0204429. doi: 10.1371/journal.pone.0204429 (PMC6152960; doi:10.1371/journal.pone.0204429)
Supplement: S1 Text — (DOCX) [file pone.0204429.s001.docx]

**Supporting Information**

**Synergy between tuberculin skin test and proliferative T cell responses to PPD or cell membrane antigens of *Mycobacterium tuberculosis* for detection of latent TB infection in a high disease-burden setting**

Suvrat Arya, Shashi Kant Kumar, Alok Nath, Prerna Kapoor, Amita Aggarwal, Ramnath Misra and Sudhir Sinha^*^

**___________________________________________________________________________**

**S1 Text. Purchased materials**

PPD was purchased from Arkray Healthcare Pvt. Ltd., Surat, India (Cat No. 18LM005-05). *Mycobacterium tuberculosis* H37Ra (MTB) was purchased from ATCC, USA (Cat No. 25177) and *Escherichia coli* (*E. coli*) was purchased from New England Biolabs, USA (Cat No. E054S). Phytohaemagglutinin (PHA) was purchased from Sigma-Aldrich, USA (Cat No.L-9017). Following fluorescent-tagged monoclonal antibodies, purchased from BD (USA), were used for phenotypic staining of T cells: CD3-FITC (UCHT1), CD4-PE Cy7 (SK3), CD8-APC (RPT-T8) and Ki67-PE (B56). Other chemicals, culture media and plasticware were purchased from BD or Sigma-Aldrich.
