# Supplementary material for: Synergy between tuberculin skin test and proliferative T cell responses to PPD or cell-membrane antigens of Mycobacterium tuberculosis for detection of latent TB infection in a high disease-burden setting
Source: PLoS One. 2018 Sep 24;13(9):e0204429. doi: 10.1371/journal.pone.0204429 (PMC6152960; doi:10.1371/journal.pone.0204429)

**S1 Fig. Protein profiles of *M. tuberculosis* and *E. coli* cell membranes determined by SDS-PAGE.** Molecular weight markers are also shown.


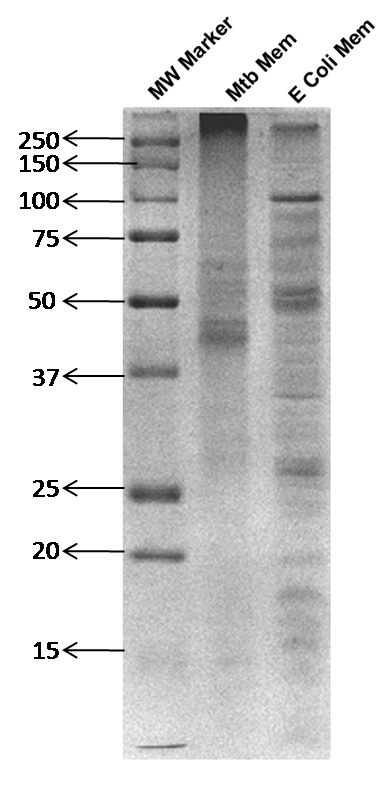

Supplement: S1 Fig — (DOCX) [file pone.0204429.s002.docx]
