# Supplementary material for: Synergy between tuberculin skin test and proliferative T cell responses to PPD or cell-membrane antigens of Mycobacterium tuberculosis for detection of latent TB infection in a high disease-burden setting
Source: PLoS One. 2018 Sep 24;13(9):e0204429. doi: 10.1371/journal.pone.0204429 (PMC6152960; doi:10.1371/journal.pone.0204429)

**S2 Fig. Gating strategy for flow cytometry.** After the lymphocyte gate [A], CD3 gating was done [B]. Among CD3+ cells, Ki67+ population was counted [C]. Similar strategy was adopted for CD4+ or CD8+ T cells.


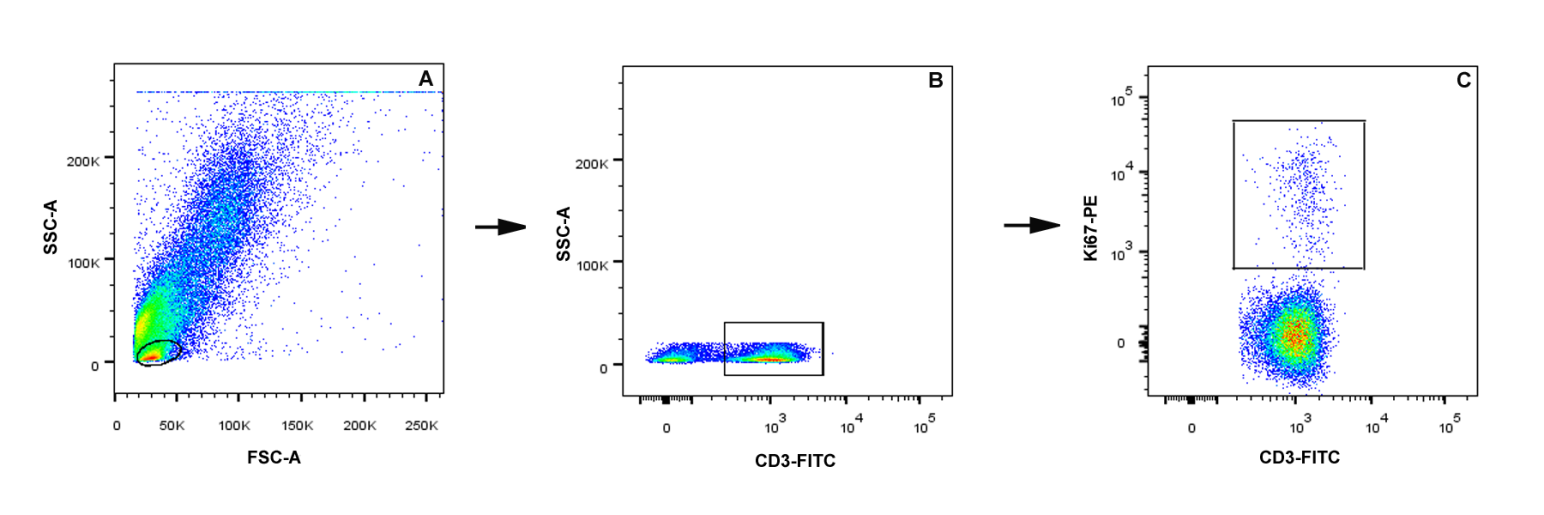

Supplement: S2 Fig — (DOCX) [file pone.0204429.s003.docx]
