# Supplementary material for: Synergy between tuberculin skin test and proliferative T cell responses to PPD or cell-membrane antigens of Mycobacterium tuberculosis for detection of latent TB infection in a high disease-burden setting
Source: PLoS One. 2018 Sep 24;13(9):e0204429. doi: 10.1371/journal.pone.0204429 (PMC6152960; doi:10.1371/journal.pone.0204429)
Supplement: S1 File — (DOCX) [file pone.0204429.s004.docx]

**S1 File. Optimization of T cell proliferation assay**

During optimization, we also tried some alternative protocols for cell fixation and permeabilization (including BD FixPerm kit) but they did not produce a neat separation of resting (Ki67-) and activated (Ki67+) T cells. Shorter incubation periods (1 or 3 days) with antigens produced weaker (than 5 days) proliferative responses, along with poor signal-to-noise discrimination (see Fig and Table below).

UNST PHA PPD MTBMem

HCW1 [D-1] 0.44 0.58 0.52 0.64

HCW1 [D-3] 0.06 10.3 0.16 0.28

HCW1 [D-5] 0.02 38.5 0.54 4.99

HCW2 [D-1] 0.48 0.69 0.44 0.45

HCW2 [D-3] 0.12 32.5 0.54 0.07

HCW2 [D-5] 0.29 79.9 1.8 1.21

**Fig and Table.** Proliferative T cell responses (%CD3+Ki67+) of 2 healthcare workers (HCW1 and 2) to RPMI1640 (UNST), T cell mitogen (PHA) and MTB antigens (PPD and MTBMem) after 1, 3 and 5 days in culture. While no appreciable responses were seen on D-1, some response, particularly to PHA, was apparent on D-3. However, D-5 responses were strongest particularly against the MTB antigens.
