# Supplementary material for: Synergy between tuberculin skin test and proliferative T cell responses to PPD or cell-membrane antigens of Mycobacterium tuberculosis for detection of latent TB infection in a high disease-burden setting
Source: PLoS One. 2018 Sep 24;13(9):e0204429. doi: 10.1371/journal.pone.0204429 (PMC6152960; doi:10.1371/journal.pone.0204429)
Supplement: S1 Table — (DOCX) [file pone.0204429.s005.docx]

S1 Table. Dataset for Fig 1A: TST responses (skin induration, mm) of HCWs categorised as OC (n=30), HC (n=8) and CTB (n=5)

OC HC CTB

0 0 0

3 10 11

6 10 14

7 14 20

9 15 35

10 19

11 20

16 30

17

2

2

2

2

3

3

4

5

6

7

7

7

10

10

10

12

13

15

15

18

20
