# Supplementary material for: Synergy between tuberculin skin test and proliferative T cell responses to PPD or cell-membrane antigens of Mycobacterium tuberculosis for detection of latent TB infection in a high disease-burden setting
Source: PLoS One. 2018 Sep 24;13(9):e0204429. doi: 10.1371/journal.pone.0204429 (PMC6152960; doi:10.1371/journal.pone.0204429)
Supplement: S2 Table — (DOCX) [file pone.0204429.s006.docx]

S2 Table. Dataset for Fig 1B: TST responses (skin induration, mm) of BCG scar positive (n=35) and BCG scar negative (n=8) HCWs.

BCG+ BCG-

0 6

3 9

7 4

10 7

11 18

16 14

17 19

2 11

2

2

2

3

3

5

6

7

7

10

10

10

12

13

15

15

20

0

10

10

15

20

30

0

14

20

35
