# Supplementary material for: Synergy between tuberculin skin test and proliferative T cell responses to PPD or cell-membrane antigens of Mycobacterium tuberculosis for detection of latent TB infection in a high disease-burden setting
Source: PLoS One. 2018 Sep 24;13(9):e0204429. doi: 10.1371/journal.pone.0204429 (PMC6152960; doi:10.1371/journal.pone.0204429)
Supplement: S3 Table — (DOCX) [file pone.0204429.s007.docx]

S3 Table. Dataset for Fig. 2B: Proliferative responses (%Ki67+) of CD3+, CD4+ and CD8+ T cells from 5 (1-5) HCWs against culture medium (MED), PHA, PPD and MTB membrane (MEM)

CD3+ CD4+ CD8+

1 2 3 4 5 1 2 3 4 5 1 2 3 4 5

MED 0.3 0.04 0.03 0.04 0.03 0.79 0.03 0.02 0.02 0.03 0 0.05 0.03 0.03 0.06

PHA 91.6 90.4 85.3 92.9 92.3 90.3 88.1 86.4 91.6 91.8 92.4 92.5 87 94 94.5

PPD 1.81 0.73 0.68 1.09 1.42 1.17 0.59 0.58 0.81 1.29 1.53 0.94 0.97 1.41 2.04

MEM 2.97 1.65 0.86 4.63 3.78 4.96 2.81 0.65 6.16 3.3 0.94 0.83 1.14 3.36 1.24
