# Supplementary material for: Synergy between tuberculin skin test and proliferative T cell responses to PPD or cell-membrane antigens of Mycobacterium tuberculosis for detection of latent TB infection in a high disease-burden setting
Source: PLoS One. 2018 Sep 24;13(9):e0204429. doi: 10.1371/journal.pone.0204429 (PMC6152960; doi:10.1371/journal.pone.0204429)
Supplement: S4 Table — (DOCX) [file pone.0204429.s008.docx]

S4 Table. Dataset for Figures 3, 4, 5A and 5B: TST (skin induration, mm) and proliferative T cell responses (%CD3+Ki67+) of HCWs (n=43) against medium (UNST), PPD and MTB membrane.

TST UNST PPD MTBMem

0 0.1 0.38 0.85

3 0.09 0.14 1.95

6 0.03 0.23 0.83

7 0.08 4.82 0.79

9 0.15 6.99 3.31

10 0.02 1.14 1.73

11 0.3 1.81 2.97

16 0.11 0.38 19.2

17 0.14 1.02 3.94

2 0.31 1.82 1.99

2 0.19 2.1 1.18

2 0.52 5.19 9.84

2 0.09 1.13 0.85

3 0.02 1.38 1.4

3 0.35 0.39 7.34

4 0.04 0.73 1.65

5 0.27 3.51 3.17

6 0.03 0.68 0.86

7 0.84 0.84 1.29

7 0.12 6.55 3.83

7 0.57 2.67 13

10 0.18 0.17 1.26

10 0.67 2.97 5.02

10 0.02 0.84 0.6

12 0.3 0.89 3.58

13 0.16 2.46 5.7

15 0.19 2.53 5.06

15 0.04 1.09 4.63

18 0.13 0.62 2.78

20 0.15 0.7 0.69

0 0.06 3.7 3.16

10 0.04 0.14 12

10 0.36 8.4 13.4

14 0.31 1.98 0.59

15 0.1 0.28 1.39

19 0.22 0.47 5.92

20 0.05 0.33 0.93

30 0.03 5.75 17.6

0 0.09 1.46 0.87

11 0.18 1.32 1.34

14 0.09 3.65 1.18

20 0.06 1.89 2.62

35 0.03 1.42 3.78
