# Supplementary material for: Synergy between tuberculin skin test and proliferative T cell responses to PPD or cell-membrane antigens of Mycobacterium tuberculosis for detection of latent TB infection in a high disease-burden setting
Source: PLoS One. 2018 Sep 24;13(9):e0204429. doi: 10.1371/journal.pone.0204429 (PMC6152960; doi:10.1371/journal.pone.0204429)
Supplement: S5 Table — (DOCX) [file pone.0204429.s009.docx]

S5 Table. Dataset for Fig 3 (inset)

PPD+ PPD-

TST+ 16 8

TST- 13 6
