# Supplementary material for: Synergy between tuberculin skin test and proliferative T cell responses to PPD or cell-membrane antigens of Mycobacterium tuberculosis for detection of latent TB infection in a high disease-burden setting
Source: PLoS One. 2018 Sep 24;13(9):e0204429. doi: 10.1371/journal.pone.0204429 (PMC6152960; doi:10.1371/journal.pone.0204429)
Supplement: S6 Table — (DOCX) [file pone.0204429.s010.docx]

S6 Table. Dataset for Fig 4 (inset)

Mem+ Mem-

TST+ 21 3

TST- 19 0
