# Supplementary material for: Synergy between tuberculin skin test and proliferative T cell responses to PPD or cell-membrane antigens of Mycobacterium tuberculosis for detection of latent TB infection in a high disease-burden setting
Source: PLoS One. 2018 Sep 24;13(9):e0204429. doi: 10.1371/journal.pone.0204429 (PMC6152960; doi:10.1371/journal.pone.0204429)
Supplement: S8 Table — (DOCX) [file pone.0204429.s012.docx]

S8 Table. Dataset for Fig 6: Proliferative T cell responses (%CD3+Ki67+) of OC (n=30, data shown in 2 rows), HC (n=8), CTB (n=5) and ATB (n=9) to MTB antigens (PPD and Mem) and mitogen (PHA).

PPD

OC 0.38 0.14 0.23 4.82 6.99 1.14 1.81 0.38 1.02 1.82 2.1 5.19 1.13 1.38 0.39

0.73 3.51 0.68 0.84 6.55 2.67 0.17 2.97 0.84 0.89 2.46 2.53 1.09 0.62 0.7

HC 3.7 0.14 8.4 1.98 0.28 0.47 0.33 5.75

CTB 1.46 1.32 3.65 1.89 1.42

ATB 1.81 0.97 0 0.84 0.31 0.56 0.2 0.21 0.32

Mem

OC 0.85 1.95 0.83 0.79 3.31 1.73 2.97 19.2 3.94 1.99 1.18 9.84 0.85 1.4 7.34

1.65 3.17 0.86 1.29 3.83 13 1.26 5.02 0.6 3.58 5.7 5.06 4.63 2.78 0.69

HC 3.16 12 13.4 0.59 1.39 5.92 0.93 17.6

CTB 0.87 1.34 1.18 2.62 3.78

ATB 2.27 2.97 0.58 0.4 0.18 1.68 0.41 0.46 2.86

PHA

OC 67.7 64.6 86.7 88.6 93.3 51.4 91.6 93.2 48.7 74.3 67.8 87.7 30.7 90.5 89.2

90.4 68.4 85.3 29 67.1 49.3 80.5 68.6 87.2 44.2 62.3 57.1 92.9 21.4 62.5

HC 87.3 76.6 69.2 84.8 80.1 92.2 81 84.2

CTB 45.6 70.9 90.9 68.5 92.3

ATB 11.8 22.4 64.1 57 10.3 94.5 13.3 29.1 27.9
